# The assessment of interhemispheric imbalance using functional near-infrared spectroscopic and transcranial magnetic stimulation for predicting motor outcome after stroke

**DOI:** 10.3389/fnins.2023.1231693

**Published:** 2023-08-16

**Authors:** Songmei Chen, Xiaolin Zhang, Xixi Chen, Zhiqing Zhou, Weiqin Cong, KaYee Chong, Qing Xu, Jiali Wu, Zhaoyuan Li, Wanlong Lin, Chunlei Shan

**Affiliations:** ^1^Department of Rehabilitation Medicine, Shanghai No.3 Rehabilitation Hospital, Shanghai, China; ^2^School of Rehabilitation Science, Shanghai University of Traditional Chinese Medicine, Shanghai, China; ^3^Center of Rehabilitation Medicine, Yueyang Hospital of Integrated Traditional Chinese and Western Medicine, Shanghai University of Traditional Chinese Medicine, Shanghai, China; ^4^Engineering Research Center of Traditional Chinese Medicine Intelligent Rehabilitation, Ministry of Education, Shanghai, China; ^5^Institute of rehabilitation, Shanghai Jiao Tong University School of Medicine, Shanghai, China

**Keywords:** stroke, transcranial magnetic stimulation, functional near-infrared spectroscopy, laterality index, interhemispheric imbalance

## Abstract

**Objective:**

To investigate changes in interhemispheric imbalance of cortical excitability during motor recovery after stroke and to clarify the relationship between motor function recovery and alterations in interhemispheric imbalance, with the aim to establish more effective neuromodulation strategies.

**Methods:**

Thirty-one patients underwent assessments of resting motor threshold (RMT) using transcranial magnetic stimulation (TMS); the cortical activity of the primary motor cortex (M1), premotor cortex (PMC), and supplementary motor area (SMA) using functional near-infrared spectroscopy (fNIRS); as well as motor function using upper extremity Fugl-Meyer (FMA-UE). The laterality index (LI) of RMT and fNIRS were also calculated. All indicators were measured at baseline(T_1_) and 1 month later(T_2_). Correlations between motor function outcome and TMS and fNIRS metrics at baseline were analyzed using bivariate correlation.

**Results:**

All the motor function (FMA-UE_1_, FMA-UE_2_, FMA-d_2_) and LI-RMT (LI-RMT_1_ and LI-RMT_2_) had a moderate negative correlation. The higher the corticospinal excitability of the affected hemisphere, the better the motor outcome of the upper extremity, especially in the distal upper extremity (*r* = −0.366, *p* = 0.043; *r* = −0.393, *p* = 0.029). The greater the activation of the SMA of the unaffected hemisphere, the better the motor outcome, especially in the distal upper extremity (*r* = −0.356, *p* = 0.049; *r* = −0.367, *p* = 0.042). There was a significant moderate positive correlation observed between LI-RMT_2_ and LI-SMA_1_ (*r* = 0.422, *p* = 0.018). The improvement in motor function was most significant when both LI-RMT_1_ and LI-SMA_1_ were lower. Besides, in patients dominated by unaffected hemisphere corticospinal excitability during motor recovery, LI-(M1 + SMA + PMC)_2_ exhibited a significant moderate positive association with the proximal upper extremity function 1 month later (*r* = 0.642, *p* = 0.007).

**Conclusion:**

The combination of both TMS and fNIRS can infer the prognosis of motor function to some extent. Which can infer the role of both hemispheres in recovery and may contribute to the development of effective individualized neuromodulation strategies.

## 1. Introduction

Stroke has a high incidence and high disability rate worldwide (Bejot et al., 2016). More than half of strokes have impaired upper limb motor function, which seriously affects the quality of life and places a huge burden on families and society (Avan et al., 2019). Currently, the recovery of upper limb motor function remains one of the challenges of post-stroke rehabilitation (Bertani et al., 2017).

Recent years have seen the rapid development of non-invasive brain stimulation technologies. Repetitive transcranial magnetic stimulation (rTMS) is considered one of the effective methods to improve upper limb motor function after stroke (van Lieshout et al., 2019; Ni et al., 2022). At present, there are two theoretical models for the clinical application of rTMS in motor rehabilitation for stroke. They are the bilateral hemispheric competition model that advocates inhibition in the unaffected hemisphere (UH) or excitation in the affected hemisphere (AH) (Lefaucheur et al., 2020) and the vicariation model that advocates excitation in residual brain area of the AH or the UH (Di Pino et al., 2014). Given that these two theoretical models are inconsistent in guiding rTMS therapy, there is no consensus on the use of excitatory or inhibitory modulation in the UH (Long et al., 2018). Understanding the progression of interhemispheric imbalance in brain activation and its contribution to motor functional recovery is important for the development of effective neuromodulation strategies (Kinoshita et al., 2019). If we know which hemisphere plays a dominant role in the process of motor function recovery, we can adopt modulatory strategies to excite that hemisphere or inhibit the contralateral hemisphere. Therefore, it is clinically important to accurately determine the interhemispheric imbalance and to develop individualized neuromodulation strategies based on it.

On the other hand, there is no uniform standard regarding the target site of rTMS stimulation for stroke patients’ motor function recovery. The most common site is the primary motor cortex (M1), because M1 is the major motor output pathway in humans (Lam et al., 2018; Lefaucheur et al., 2020). In addition, studies suggested that the secondary motor cortical areas, including the premotor cortex (PMC) and supplementary motor area (SMA), can also be used as modulatory targets. The PMC has fiber connections to M1 in both the ipsilateral and contralateral hemispheres. When the lesion is large over M1 area, the PMC can function instead of M1 (Buetefisch, 2015; Plow et al., 2015). Similarly, functional connections exist between SMA and M1 and SMA are also involved in corticospinal projections that may facilitate motor recovery after stroke (Matsunaga et al., 2005; Diao et al., 2017). Therefore, an in-depth understanding of the changes in the motor cortex during function improvement facilitates us to understand the mechanisms of functional recovery.

As a neuromodulation technique, transcranial magnetic stimulation (TMS) is not only a treatment therapy but also an assessment tool (Groppa et al., 2012). Single-pulse TMS assessment metrics such as resting motor threshold (RMT), and motor evoked potential (MEP) can evaluate the excitability of corticospinal motor neurons, and the functional integrity of the corticospinal tract (CST) (Andrews et al., 2022). It has been demonstrated indicators by TMS can be used as valid biomarkers to assess the recovery of neurological function (Kelley et al., 2014). Besides, another study measured RMT by TMS and calculated the laterality index (LI) of cortical excitability to confirm asymmetric functional changes in the cerebral hemispheres after stroke (Di Lazzaro et al., 2016). Studies have shown that the success of motor recovery after stroke is significantly determined by the direction and extent of cortical excitability changes in both hemispheres (Stinear et al., 2015; Veldema et al., 2021). Understanding the contribution of either cortical hemisphere to motor recovery may facilitate the development of effective individualized rehabilitation strategies (Kumar et al., 2016). So, the TMS assessment may identify the altered interhemispheric imbalance and guide selection of appropriate modulation protocol for stroke patients.

In addition to TMS, functional near-infrared spectroscopy (fNIRS) is a neuroimaging method for assessing brain function (Delorme et al., 2019). fNIRS can detect activation patterns in the cerebral motor cortex, reflecting changes in neural remodeling during the recovery of motor function after stroke (Huo et al., 2019). It has been shown that cerebral hemodynamic activity reflected by fNIRS can be a reliable neurobiomarker for the assessment of limb motor dysfunction in stroke patients (Wang et al., 2023). It is well known that stroke-induced motor deficits are associated with an interhemispheric imbalance of motor activation (Cunningham et al., 2015; Tang et al., 2015; Kinoshita et al., 2019). As motor function is restored, the balance of interhemispheric activation in the motor-related cortex changes (Tang et al., 2015). Then, a study showed that assessing cortical activation asymmetry by fNIRS can help predict the response to rehabilitation treatment (Tamashiro et al., 2019). Thus, fNIRS also promises to be a convenient technique for investigating the neural mechanisms underlying the dysfunction, which will deepen our understanding of stroke rehabilitation and potentially translate this knowledge to improve the effectiveness of rehabilitation interventions.

Although TMS and fNIRS assessment techniques have been used in clinical research, they also have certain limitations. fNIRS is able to detect a wide range of cortical areas, but it is limited by spatial resolution, which prevents it from detecting deep brain regions (Ni et al., 2022). In contrast, TMS measures the corticospinal excitability by evoking MEP in the primary motor cortical (M1). It only reflects the functional integrity of the cortical downstream pathways emanating from M1, but compensates for the inability of fNIRS assessment to access deep brain motor pathways. The combined use of both methods will help us to gain insight into the changes in brain function during motor recovery after stroke. Therefore, we conducted a longitudinal, multimodal study using the clinical scale, TMS, and fNIRS measures to find the relationship between motor function recovery and interhemispheric imbalance changes.

## 2. Materials and methods

### 2.1. Participants

The present study was approved by the ethics committee at Shanghai No.3 Rehabilitation Hospital (ethics No. SH3RH-2021-EC-012), and was registered in the China Clinical Trial Registration Center (registration No. ChiCTR2200057378). All patients were informed about the nature of this study.

Thirty-one first-ever stroke patients (age 65.42 ± 10.15 years) participated in this study. The participants with stroke in this study were recruited from Shanghai No.3 Rehabilitation Hospital. The basic information of participants was presented in Table 1. Criteria for selecting the subjects were as follows: (1) first-ever stroke, (2) age of 30 and 80 years, (3) clear consciousness and stable vital signs, (4) no cognitive impairment (mini-mental state examination score ≥ 24 points). The exclusion criteria were: (1) contraindications to TMS (Najib and Horvath, 2014), (2) having visual and hearing impairment and cannot cooperate to complete the trials, (3) having severe heart, liver, or kidney dysfunction or malignancy, (4) other neurological diseases.

**Table 1:** Demographic data and clinical history of the patients.

Patient	Sex	Age (years)	Stroke type	AH	Stroke duration (days)	BI	FMA-UE_1_
1	F	59	Infarction	L	95	90	17
2	M	47	Infarction	L	26	90	54
3	M	69	Infarction	L	178	25	6
4	M	66	Infarction	L	47	45	10
5	M	66	Infarction	L	81	45	12
6	M	77	Infarction	L	441	55	41
7	M	66	Infarction	L	165	40	4
8	F	72	Infarction	L	32	45	37
9	M	38	Infarction	L	10	95	64
10	F	76	Infarction	L	209	75	10
11	M	66	Infarction	R	62	40	25
12	M	66	Infarction	R	96	40	27
13	M	66	Infarction	R	80	45	32
14	F	66	Infarction	R	35	40	0
15	M	57	Infarction	R	16	40	0
16	M	48	Hemorrhage	R	68	55	6
17	M	78	Infarction	R	59	90	44
18	M	64	Infarction	R	68	30	6
19	F	61	Infarction	R	9	35	8
20	F	74	Infarction	R	101	65	35
21	F	79	Infarction	L	102	30	16
22	M	73	Infarction	L	153	60	15
23	M	61	Infarction	L	182	45	26
24	M	66	Infarction	R	177	60	21
25	M	42	Hemorrhage	L	29	45	0
26	M	73	Infarction	L	32	60	19
27	F	72	Hemorrhage	R	88	45	9
28	M	72	Infarction	R	20	65	40
29	M	72	Hemorrhage	R	224	55	40
30	F	65	Infarction	R	51	70	22
31	M	71	Hemorrhage	R	63	60	36

M, male; F, female; L, left; R, right; AH, affected hemisphere; BI, Barthel index; FMA-UE_1_, Fugl-Meyer assessment for the upper extremity score at baseline.

### 2.2. Experimental design

In this study, patients underwent clinical, neurophysiological, and neuroimaging assessments. The clinical assessment was performed using FMA-UE. The neurophysiological assessment was performed using RMT for corticospinal excitability. The neuroimaging assessment was performed using fNIRS to assess changes in motor cortical activation. The assessment metrics were labeled as 1 at baseline and 2 in 1 month later, such as FMA-UE_1_ and FMA-UE_2_. All participants received conventional medical treatments and rehabilitative therapies during participation. The conventional rehabilitative therapies include physiotherapy and occupational therapy.

### 2.3. Assessments and procedures

#### 2.3.1. Clinical assessment

FMA-UE was used to quantify the initial deficit and to follow up on the recovery of voluntary movements of the paralyzed limb after 1 month. All clinical assessments were performed by a blinded specialized assessor at baseline and 1 month later. FMA-UE is a measure of upper extremity motor function for stroke patients with a total score of 66, which means that the lower the score, the more severe the degree of impairment (Gladstone et al., 2002). The distal portion of the tested upper limb in FMA-UE was recorded as FMA-d, while the proximal portion was recorded as FMA-p.

#### 2.3.2. TMS assessment

**Figure 1:**
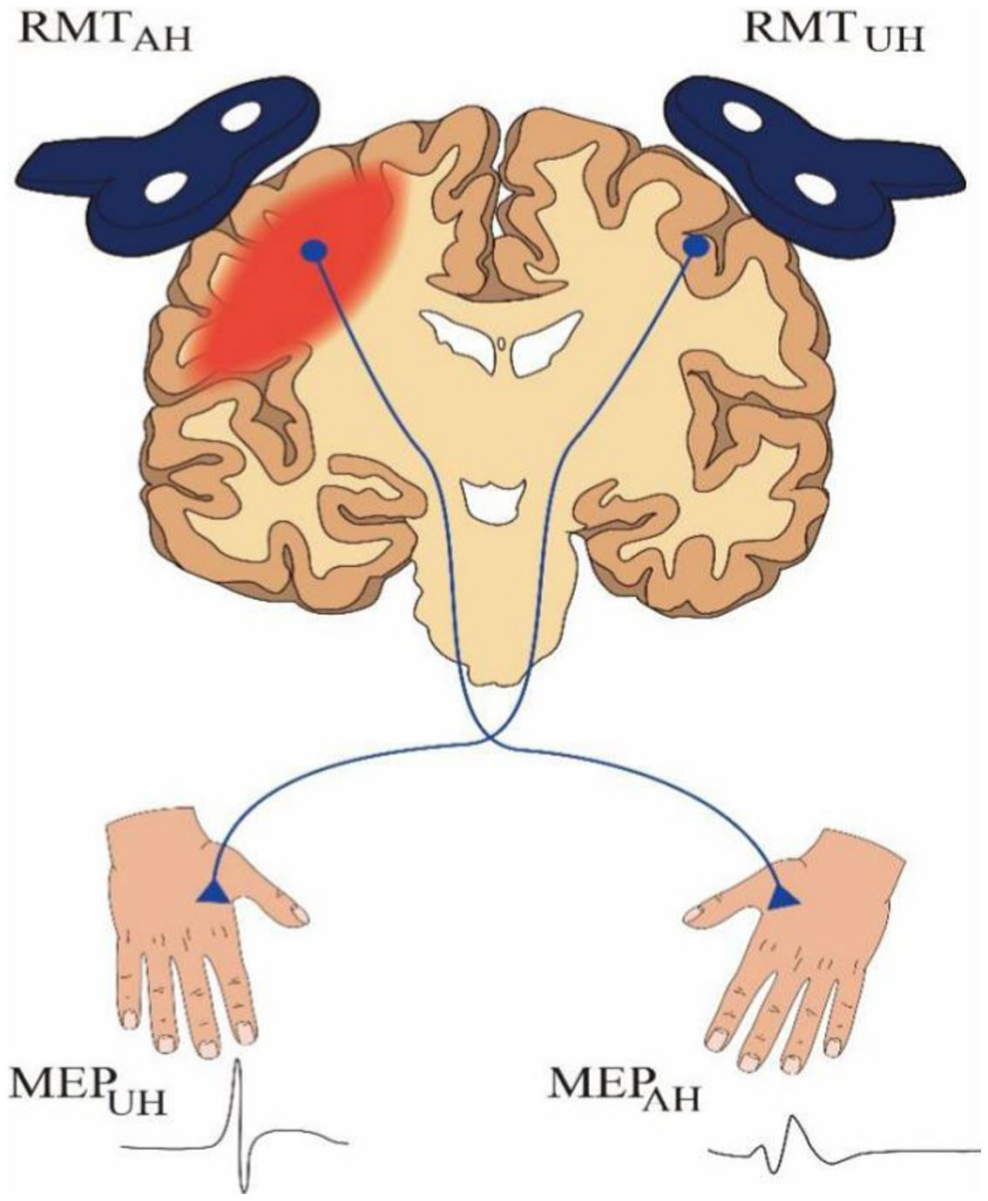
A schematic representation of TMS assessment. RMT, resting motor threshold; MEP, motor evoked potential; AH, affected hemisphere; UH, unaffected hemisphere.

All subjects were evaluated with TMS which was carried out by M-100 Ultimate Pulsed magnetic Stimulation Device (Shenzhen Yingchi Technology Co., Ltd., Shenzhen, China). The patients were asked to sit in a relaxed position, and the skin on which the electrodes were attached was defatted with 95% alcohol cotton balls. The recording electrode was located in the muscle belly of the first dorsal interosseous (FDI), the reference electrode was located in the tendon of FDI, and the ground electrode was located in the forearm. Detection was performed in a single-pulse stimulation mode. The evaluator held a figure-of-eight-shaped coil with BY90A model to deliver TMS to the motor cortex. The maximum magnetic field change rate on the coil surface was 16.09kT/s, the peak stimulus intensity was 1 T, the pulse rise time was 62.0 μs, and the bidirectional wave unilateral pulse width was 200 μs. The optimal location of the M1 was first found according to the international 10/20 EEG positioning system, and then the coil was moved in small steps in the hand region of M1 until the position where maximal MEPs were consistently obtained was found. The coil was placed on the scalp at this location with handles pointing backward and rotated approximately 45° from the midline. TMS intensity was expressed as a percentage of maximum stimulator output (%MSO). The minimum TMS intensity with MEP which was elicited in the contralateral FDI greater than 50 μV for at least 5 of 10 consecutive single-pulse stimuli was recorded as RMT. RMT was measured on both hemispheres separately, inducing MEP in the contralateral FDI (see Figure 1). For patients without evoked MEPs, RMT in the AH was defined as 110 %MSO (Kemlin et al., 2019).

#### 2.3.3. fNIRS assessment

**Figure 2:**
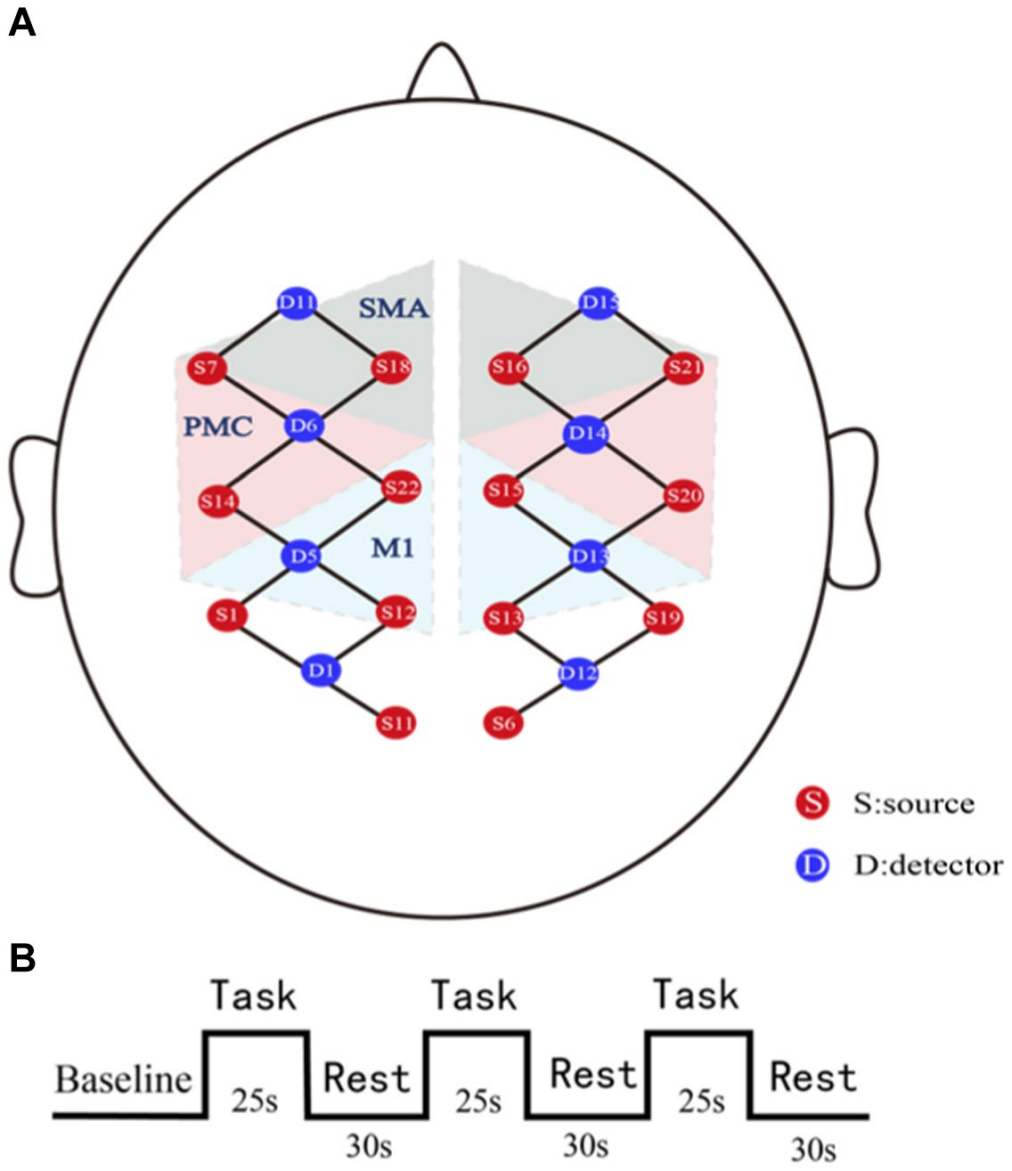
Schematic representations of fNIRS assessment. **(A)** fNIRS channels and region of interests (ROIs) map. SMA, supplementary motor area; PMC, premotor cortex; M1, the primary motor cortex. **(B)** Block design paradigm. Each task block lasted 25 s and each rest lasted 30s. The 10s pre-task period was used as the baseline.

The fNIRS data acquisition was performed using the NirSmart system (Danyang Huichuang Medical Equipment Co., Ltd., China) with a sampling rate of 11 Hz. Optical signals of two different wavelengths (730 nm and 850 nm) can be recorded in the continuous waveform in this system. There are 14 light sources and 8 detectors on the acquisition cap, with a total of 26 channels. The region covered by the probe involves the sensorimotor areas of the bilateral cerebral cortex. We predefined the regions of interest (ROIs), including M1, SMA, and PMC (See Figure 2A).

In this study, fNIRS data in the task state were collected. All patients were asked to sit quietly and relaxed. The assessor would communicate the entire task flow with the patients in advance and ask them not to speak during the task. The task paradigm was block-designed, which consists of alternating 3 grasping tasks and 3 rests. Each task block lasted 25 s and each rest lasted 30s (See Figure 2B). During the task period, patients grasped actively or passively (if the patients were unable to grasp actively, the assessor helped the patients to grasp passively) (Du et al., 2019).

### 2.4. Data analysis

#### 2.4.1. TMS data processing and analyses

To evaluate the hemispheric asymmetry of motor cortex excitability, we calculated the interhemispheric LI of the RMT. The LI of RMT was calculated as Formula (1).

(1) $$\mathrm{LI}\left(\mathrm{RMT}\right)=\frac{{\mathrm{RMT}}_{\mathrm{AH}}-{\mathrm{RMT}}_{\mathrm{UH}}}{{\mathrm{RMT}}_{\mathrm{AH}}+{\mathrm{RMT}}_{\mathrm{UH}}}$$

LI (RMT) represents the imbalance of corticospinal excitability in both hemispheres. Thus, a positive value indicates higher corticospinal excitability in the UH. The greater the difference from 0, the higher the degree of imbalance between the cerebral hemispheres (Di Lazzaro et al., 2016).

RMT was also expressed as a ratio (*R* = RMT_AH_/RMT_UH_) (Kemlin et al., 2019). The value after 1 month (recorded as R2) minus the baseline (recorded as R1) obtained the difference of R, i.e., d(*R*). d(*R*) > 0 indicates more changes in corticospinal excitability in the UH and d(*R*) < 0 indicates more changes in corticospinal excitability in the AH (Patel et al., 2020; de Freitas et al., 2022).

#### 2.4.2. fNIRS data preprocessing and analyses

The fNIRS data were preprocessed using the Homer 2.0 toolkit under the MatlabR2013a operating environment. Data pre-processing includes conversion of the original signals, identification of artifacts and noise, as follows: data conversion to convert the raw signal into light intensity; identification of artifacts and correction; band-pass filtering of noise in the range of 0.01–0.1 Hz to eliminate possible respiratory and heart rate interference; conversion of the filtered light intensity into oxyhemoglobin (HBO) level according to the modified Beer-Lamber law. We analyze the HBO level because it is reliable and sensitive to changes in cerebral blood flow (Kinoshita et al., 2019).

The preprocessed data are imported into NirSpark analysis software. Firstly, the three segment blocks of each data were averaged according to the mark to obtain accurate and stable data. The HBO concentration within 5 s before task onset was used as the baseline. The average HBO concentration during task performance minus the baseline concentration was the relative change in HBO concentration (∆HBO). The ∆HBO on the corresponding channel of the ROI on each hemisphere was extracted and the LI was calculated to assess the hemispheric imbalance to determine the relative hemispheric dominance induced by the grasping task on the hemiplegic side (Borrell et al., 2023). The LI of ∆HBO was calculated as Formula (2). According to the following published literature in the previous manuscript (Borrell et al., 2023), the absolute value (ABS) in the denominator of the formula would prevent possible zero value.

(2) $$\begin{array}{c} \mathrm{LI}\left(\Delta \mathrm{HBO}\right)=\frac{\Delta {\mathrm{HBO}}_{\mathrm{AH}}-\Delta {\mathrm{HBO}}_{\mathrm{UH}}}{ABS\left(\Delta {\mathrm{HBO}}_{\mathrm{AH}}\right)+ABS\left(\Delta {\mathrm{HBO}}_{\mathrm{UH}}\right)} \end{array}$$

LI (∆HBO) value ranges from −1 to 1, and it reveals which hemisphere experiences a larger change during the task. Negative LI (∆HBO) values indicate UH dominant activity, while positive LI (∆HBO) values indicate AH dominant activity (Borrell et al., 2023). Thus, the LI (∆HBO) value of “−1” indicates complete UH dominance, and the LI (∆HBO) value of “+1” indicates complete AH dominance. In this study, we calculated the LI of M1, SMA, PMC, and M1 + SMA + PMC.

### 2.5. Statistics analysis

Statistical analyses were performed with SPSS V.24.0 software. Behavioral data at baseline and 1 month later were tested using a paired *t*-test if they conformed to a normal distribution, otherwise, a nonparametric test was used. We evaluated the bivariate relation between the neuroimaging metrics and TMS metrics. To identify functionally relevant metrics, we calculated the correlation between FMA-UE and TMS (LI-RMT) and neuroimaging metrics (LI-M1, LI-SMA, LI-PMC). To explore the correlation between the indicators in the case of recovery dominated by different cerebral hemispheres (AH or UH), we additionally grouped the d(*R*) > 0 and d(*R*) < 0 groups according to d(*R*) and performed correlation analyses. Pearson’s correlation test was used if a normal distribution was consistent, and nonparametric Spearman’s correlation test was used otherwise. Data conforming to the normal distribution are denoted by mean ± SD, otherwise, M (P25, P75). All data used a two-sided calibration with a test level of α = 0.05. According to the previous study (Prion and Haerling, 2014), the interpretation of correlation coefficients are as follows: 0 to ±0.20 is negligible, ±0.21 to ±0.35 is weak, ±0.36 to ±0.67 is moderate, ±0.68 to 0.90 is strong, and ± 0.91 to ±1.00 is considered very strong.

## 3. Results

All 31 subjects completed two assessments at baseline and 1 month later. At the TMS assessment, a total of 20 subjects were unable to induce the MEP at baseline and 19 subjects were unable to induce the MEP 1 month later. In addition, 23 subjects required assistance to complete the grasping task at the fNIRS assessment.

### 3.1. Metrics of interhemispheric imbalance and clinical assessment of motor function

#### 3.1.1. Relationship between motor function and hemispheric asymmetry of corticospinal excitability

For all patients, motor function was significantly improved 1 month later compared to baseline (*p* < 0.05).

**Figure 3:**
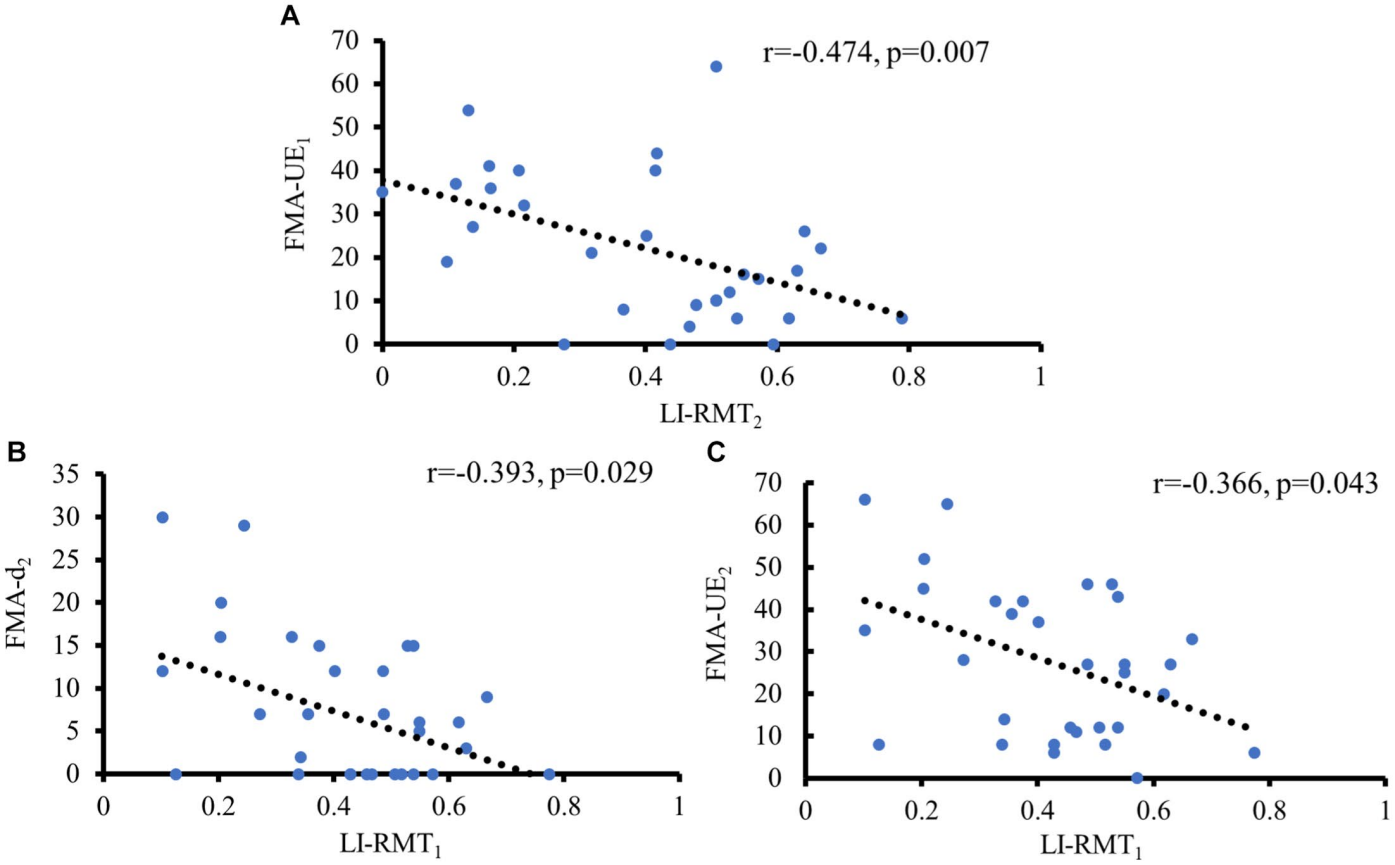
Significant correlations of motor function with LI- RMT. **(A)** Negative correlation between FMA-UE at baseline and LI-RMT at 1 month later; **(B)** Negative correlation between LI-RMT at baseline and FMA-UE at 1 month later; **(C)** Negative correlation between LI-RMT at baseline and FMA-d at 1 month later.

There was a significant moderate negative correlation between LI-RMT_1_ and FMA-UE_2_ as well as FMA-d_2_ (*r* = −0.366, *p* = 0.043; *r* = −0.393, *p* = 0.029), which means the higher the corticospinal excitability of the AH, the better the motor outcome of the upper extremity, especially in the distal upper extremity. Additionally, a moderate negative correlation was observed between LI-RMT_2_ and FMA-UE_1_ (*r* = −0.474, *p* = 0.007) (see Figure 3).

#### 3.1.2. Relationship between motor functional recovery and interhemispheric imbalance in motor cortical activity

**Figure 4:**
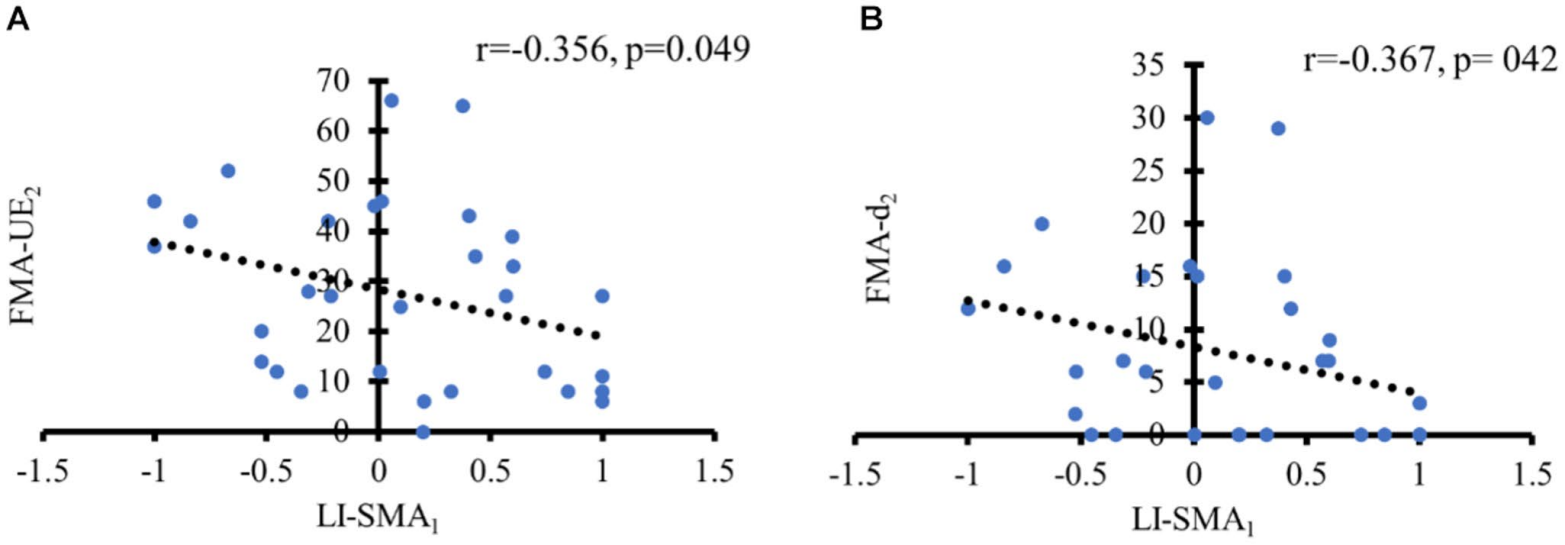
Significant correlations of motor function with LI of motor cortical activity. **(A)** Negative correlation between LI-SMA at baseline and FMA-UE at 1 month later; **(B)** Negative correlation between LI-SMA at baseline and FMA-d at 1 month later.

For all patients, LI-SMA_1_ exhibited a significant moderate negative association with both FMA-UE_2_ and FMA-d_2_ (*r* = −0.356, *p* = 0.049; *r* = −0.367, *p* = 042), which means the greater the activation of the SMA of the UH, the better the motor outcome, especially in the distal upper extremity (see Figure 4).

#### 3.1.3. Relationship between motor function and combined fNIRS and TMS metrics

**Figure 5:**
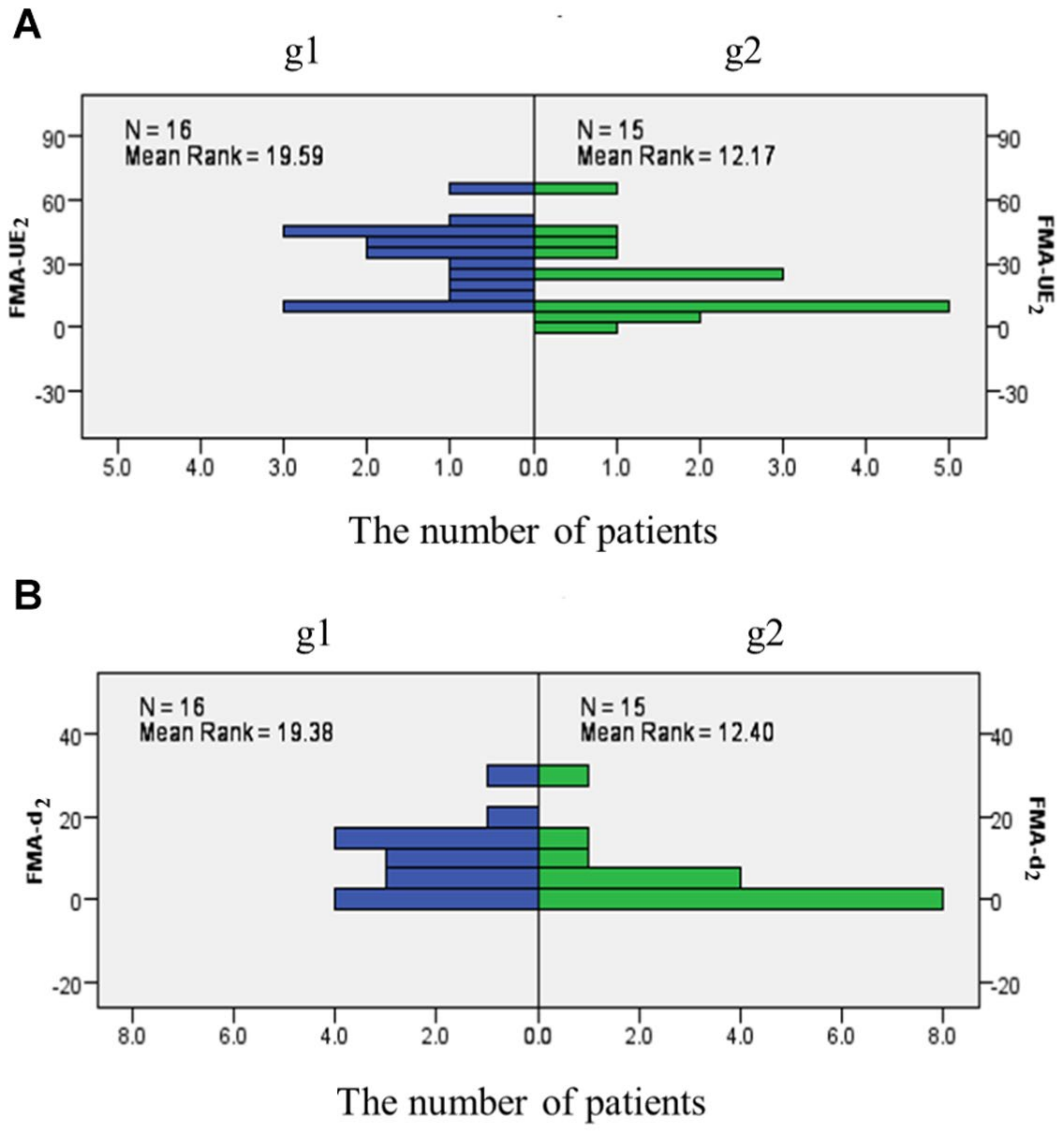
The frequency of FMA-UE_2_ and FMA-d_2_ in different groups. **(A)** The frequency distribution map as FMA-UE at 1 month later; **(B)** The frequency distribution map as FMA-d at 1 month later.

We calculated the mean value of LI-SMA_1_ + LI-RMT_1_. All patients were grouped according to the obtained mean, with one group being < mean (named g1) and the other group being > mean (named g2). There was a significant difference between g2 and g1. Compared with the g2 at the same time point, the function score of FMA-UE_2_ and FMA-d_2_ increased faster in g1 (*p* = 0.023 and p = 0.029, respectively), as shown in Table 2 and Figure 5. Both of these imply that the two states of SMA and M1 of the patient at baseline could predict future functional recovery.

**Table 2:** Comparison between g1 and g2 with FMA-UE and FMA-d at different times.

Group	FMA-UE_1_	FMA-UE_2_	FMA-d_1_	FMA-d_2_
	M (P25, P75)	M (P25, P75)	M (P25, P75)	M (P25, P75)
g1 (*n* = 16)	24 (11.3,40)	36 (15.5, 45.8)	6.5 (0,14.5)	12 (3.0, 15.8)
g2 (*n* = 15)	12 (6, 26)	12 (8.0, 33.0)	0 (0, 7.0)	0 (0, 7.0)
*Z*	−1.622	−2.278	−1.707	−2.187
*p*	0.105	0.023*	0.088	0.029*

*g*1, the group of the value from LI-SMA_1_ + LI-RMT_1_ < mean; *g*2, the group of the value from LI-SMA_1_ + LI-RMT_1_ > mean; *Significant median difference at *p* < 0.05.

### 3.2. Relationship between TMS and fNIRS measure of interhemispheric balance

**Figure 6:**
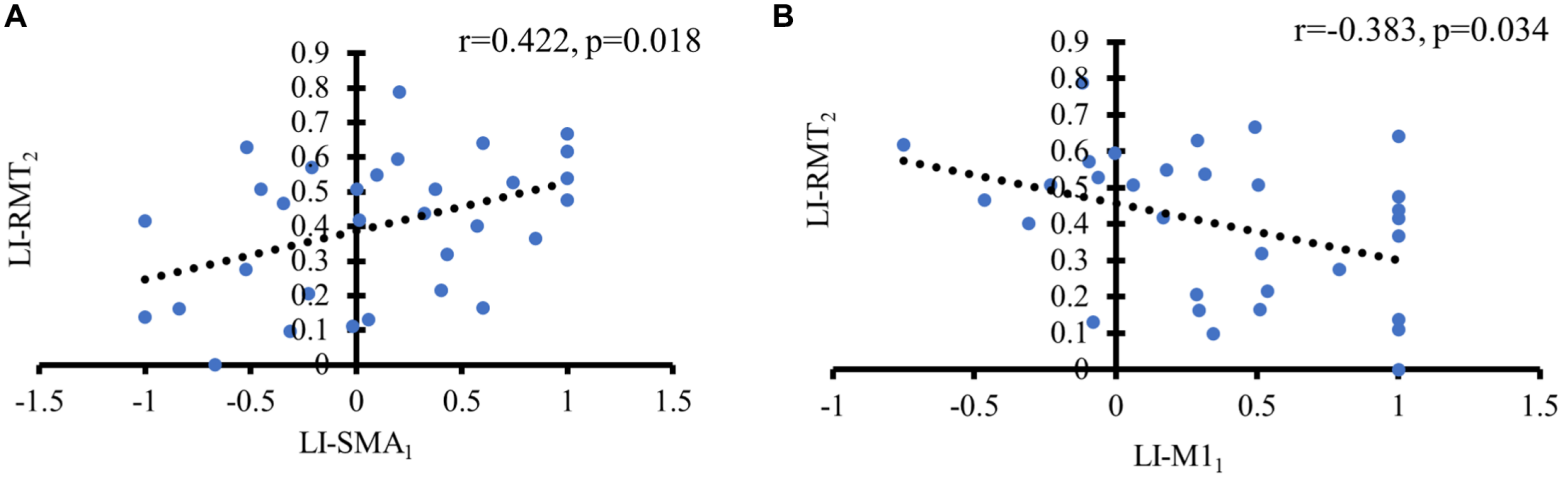
Significant correlations of TMS with fNIRS metrics. **(A)** Positive correlation between LI-SMA at baseline and LI-RMT at 1 month later; **(B)** Negative correlation between LI-M1 at baseline and LI-RMT at 1 month later.

For all patients, there was a significant moderate positive correlation observed between LI-RMT_2_ and LI-SMA_1_ (*r* = 0.422, *p* = 0.018). Additionally, LI-RMT_2_ exhibited a negative association with LI-M1_1_ (*r* = −0.383, *p* = 0.034) (see Figure 6).

### 3.3. Correlation of motor outcome based on interhemispheric asymmetry of corticospinal excitability

**Figure 7:**
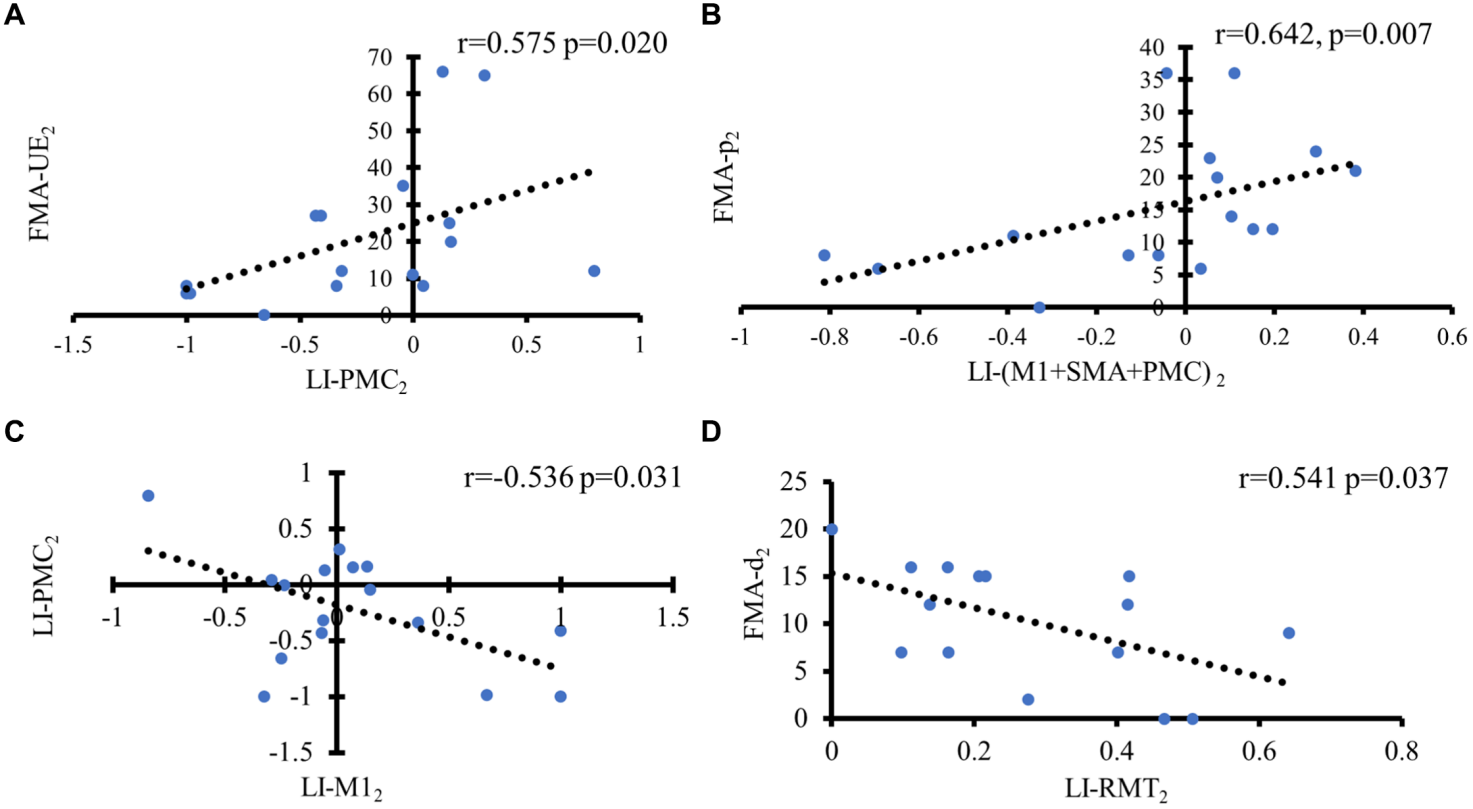
Correlation analysis of motor outcome in different groups based on hemispheric asymmetry of corticospinal function. In the d(*R*) > 0 group, **(A)** Positive correlation between LI of PMC and FMA-UE at 1 month later; **(B)** Positive correlation between LI of M1 + SMA + PMC and FMA-p at 1 month later; **(C)** Negative correlation between LI-M1 and LI-PMC at 1 month later. In the d(*R*) < 0 group, **(D)** Negative correlation between FMA-d and LI-RMT at 1 month later.

In the d(*R*) > 0 group, LI-PMC_2_ exhibited a significant positive association with FMA-UE_2_ (*r* = 0.575, *p* = 0.020). LI-(M1 + SMA + PMC)_2_ exhibited a positive association with FMA-p_2_ (*r* = 0.642, *p* = 0.007). There was a negative relation between LI-M1_2_ and LI-PMC_2_ (*r* = −0.536, *p* = 0.031) (see Figures 7A–C).

In the d(*R*) < 0 group, FMA-d_2_ had a negative relation with LI-RMT_2_ (*r* = −0.541, *p* = 0.037). However, no significant associations were found between fNIRS metrics and TMS metrics in the d(*R*) < 0 (see Figure 7D).

## 4. Discussion

It is known that the anatomic structure of the left and the right brain is generally symmetrical (Tang et al., 2015). A functional balance exists between the two hemispheres of the healthy brain, regulated by interhemispheric inhibition. This balance is disrupted after the onset of stroke (Shen et al., 2022). However, the role of the motor cortex of both hemispheres in the recovery of motor function remains controversial, especially the UH (Ni et al., 2022). Some studies show that activation of the UH increases interhemispheric inhibition in the AH (Dionisio et al., 2018; Lefaucheur et al., 2020), while others suggest that activation of the UH plays a compensatory role for the inactivation of the AH (Bradnam et al., 2012; Bertolucci et al., 2018). Therefore, predicting motor function outcomes based on the asymmetry of cortical excitability and motor cortical activation is important for developing rehabilitation programs.

The RMT measured by TMS is a standard measure of corticospinal excitability, and the ∆HBO measured by fNIRS is used to assess brain activation (Lee et al., 2019; Badran et al., 2020). To date, no combination of the two assessment methods to assess the imbalance in brain asymmetry has been reported. Our study is the first to combine TMS and fNIRS for investigating motor function outcome after stroke. We calculated the LI of RMT and ∆HBO, which reflects the asymmetry between the cerebral hemispheres, and investigated its relationship with motor function recovery. Exploring the underlying neural mechanisms of functional recovery after stroke will help us to develop new rehabilitation interventions.

In the current study, the patients had recovery of hemiplegic upper limb motor function after one month. It may have been a spontaneous neurological recovery or benefited from conventional rehabilitation. In these patients, those who initially had better motor function subsequently also had corticospinal excitability lateralized to the AH. Similarly, the more corticospinal excitability was lateralized to the AH at baseline, the better motor outcome after a month of recovery time. The result is consistent with the common pattern of motor function recovery. The lateralization of corticospinal excitability reflected by LI-RMT to the AH indicates that the functional integrity of the affected CST is not completely disrupted. The CST in the AH plays an important role in motor function recovery in the hemiplegic limb. It has been shown that the surviving neurons on the AH contribute to axonal remodeling of the CST, which promotes motor recovery after stroke (Okabe et al., 2017). Our result suggests that functional integrity of the CST measured by TMS-induced MEP helps predict motor function outcomes, which is consistent with previous studies suggesting that MEP deficits in the AH are associated with poorer motor recovery after stroke (Chen et al., 2023).

However, TMS assessment is obtained by evoking MEP in M1. It can only reflect the excitability of the corticospinal pathway emanating from M1. Compared to TMS, fNIRS can measure the activation of the entire motor cortex. Our study suggests that combining fNIRS with TMS provides complementary information superior to that of imaging methods in isolation. We found that the patients with SMA activation lateralized to the UH at baseline had better motor outcomes.

The SMA is a secondary motor area that plays a pivotal role in complex hand movements (Shirota et al., 2012). SMA and M1 both have a direct influence on force production during fine manual motor tasks (Entakli et al., 2014). A previous study using fMRI also found a correlation between task-related brain activation patterns and final motor status. They found that greater brain activation in the SMA at baseline was associated with better motor outcomes after stroke (Du et al., 2018). This may be specific recruitment of SMA to provide motor control in order to produce motion output during the motor task. Previous studies found that better motor outcomes were associated with higher baseline activation in bilateral SMA (Du et al., 2018; Xu et al., 2021), whereas we found that it was SMA activation lateralized to the UH that led to better motor outcomes, especially in distal upper extremity motor function. Our data support the critical role of SMA activation lateralized to the UH in stroke recovery.

Furthermore, the relationship between the lateralization of corticospinal excitability in TMS and the lateralization of motor cortical activation in fNIRS was analyzed. At baseline, if M1 activation was lateralized to the AH, the improvement in motor function was accompanied by lateralization of corticospinal excitability to the AH. It is consistent with the model that motor functional recovery after stroke is dominated by M1 neural remodeling in the AH (Buetefisch, 2015; Yang et al., 2021). If SMA activation was lateralized to the UH, recovery of motor function was accompanied by lateralization of corticospinal excitability to the AH. A previous study has shown that improved motor function after stroke might be highly correlated with the functional connectivity of the ipsilesional M1 to the contralateral SMA (Chen et al., 2021). In this case, the SMA in the AH may play a compensatory role.

Our study shows that a multimodal model combining fNIRS, TMS, and clinical assessment predicts motor outcomes after stroke. It may have important clinical implications in guiding neuromodulation rehabilitation strategies. Previous studies have shown that inhibitory or facilitative rehabilitation techniques can be used based on the imbalance in interhemispheric cortical excitability (Du et al., 2019; Veldema et al., 2021). It is well known that M1 is the major motor output pathway for motor control (Lam et al., 2018) and is also the most commonly used stimulation target for neuromodulation (Hallett et al., 2017; Lefaucheur et al., 2020). However, our results show that activation of SMA lateralized to the UH is also associated with good motor outcome. Besides, the improvement in motor function was most significant when both the corticospinal excitability predominantly in the AH (LI-RMT_1_) and SMA activation predominantly in the UH (LI-SMA_1_) conditions were met at baseline (Figure 3). Thus, it is reasonable to assume that the best motor outcome may be obtained when stimulating the M1 of the AH is added with excitatory stimulation of the SMA of the UH. We hypothesize that the SMA of the UH may play a compensatory role in motor recovery, especially in the distal limb motor. So, the SMA may also be a suitable target for rTMS stimulation to develop specific rehabilitation methods. However, the underlying mechanism needs further clinical study.

In addition, we calculated R-values using the RMT measured by TMS, which reflects the interhemispheric imbalance of corticospinal excitability. According to the difference in R-values before and after one month, the patients were divided into two groups. In the d(*R*) < 0 group, corticospinal excitability changes were dominated by AH, while in the d(*R*) > 0 group, corticospinal excitability changes were dominated by UH. In the group dominated by the AH, motor outcome of the distal upper limb was positively correlated with altered AH corticospinal excitability. In other words, good motor function of the distal upper limb depended on the degree of recovery of CST function emanating from the AH.

Notably, in the group dominated by UH corticospinal excitability, motor outcomes in the upper limb as well as in the proximal were associated with AH-dominated PMC activation. We hypothesize that the CST emanating from AH is severely impaired in this group, and therefore the corticospinal excitability emanating from UH is dominant. This also implies that the anterior CST emanating from the UH has enhanced conduction signals, which can innervate the ipsilateral hemiplegic limb. Moreover, this compensatory neural pathway innervates more proximal muscles than distal muscles (Wang et al., 2019). On the other hand, it has been demonstrated that PMC has fiber connections to M1 in both the ipsilateral and contralateral hemispheres and that PMC in AH can function instead of M1 (Dum and Strick, 1991; Kantak et al., 2012). Meanwhile, our results show that AH-dominant PMC activation is positively correlated with UH-dominant M1, suggesting that recovery in the motor may be accompanied by enhanced functional connectivity between these two brain areas. It also plausibly explains the positive correlation between motor outcome and AH-dominated PMC activation observed in the proximal of the upper limb in this group. We demonstrate from the interhemispheric imbalance of corticospinal excitability combined with the lateralization of cortical activation that multiple mechanisms may be involved in the process of motor function recovery. These may include the compensation of the PMC within the AH and the compensating effect of the UH.

The structure of the brain is complex, and the connections between brain regions are also variable. Our study suggests that combining fNIRS with TMS provides complementary information superior to that of imaging methods in isolation. It helps to deepen our understanding of brain diseases and provides valuable information for further exploration of neural mechanisms.

However, due to the limited sample size in our study, stratified analysis could not be performed. It is the limitation of our present study. Future studies will need to enroll more post-stroke patients to conduct stratified analysis by disease duration, disease severity, and disease type.

## 5. Conclusion

In conclusion, the present study provided evidence that the interhemispheric imbalance between corticospinal excitability and motor cortex activation can be a biomarker for predicting motor recovery. The combined assessment of TMS and fNIRS can infer the role of both hemispheres in recovery and contribute to the development of effective individualized neuromodulation strategies. Further studies should include more participants with stroke to obtain a reliable relationship between these features and motor function state.

## Data availability statement

The original contributions presented in the study are included in the article/supplementary material, further inquiries can be directed to the corresponding authors.

## Ethics statement

The studies involving human participants were reviewed and approved by the Ethics Committee of Shanghai No.3 Rehabilitation Hospital. The patients/participants provided their written informed consent to participate in this study.

## Author contributions

SC, XZ, and XC are the co-first authors of this paper. They participated in all studies, analyzed the data, drafted and finalized the manuscript. CS and WL are co-corresponding authors of this paper. They contributed to conception and design of the study, supervised the progression, and revised the manuscript. ZZ consulted on data analysis. KC coordinated data collection and performed the statistical analysis. WC and JW collected and sorted out the related materials. QX and ZL were involved in the recruitment of participants. All authors contributed to the manuscript revision, read and approved the submitted version.

## Funding

This work was supported by National Natural Science Fund (Grant No. 82272612), Shanghai Jing’an District Health Research Project (Grant No. 2021MS19), Shanghai Clinical Research Center for Rehabilitation Medicine (21MC1930200) and Key Subjects Construction Program of the Health System in Jing’an District (Grant No. 2021PY04).

## Conflict of interest

The authors declare that the research was conducted in the absence of any commercial or financial relationships that could be construed as a potential conflict of interest.

## Publisher’s note

All claims expressed in this article are solely those of the authors and do not necessarily represent those of their affiliated organizations, or those of the publisher, the editors and the reviewers. Any product that may be evaluated in this article, or claim that may be made by its manufacturer, is not guaranteed or endorsed by the publisher.

## References

[ref1] Andrews S. C. Curtin D. Coxon J. P. Stout J. C. (2022). Motor cortex plasticity response to acute cardiorespiratory exercise and intermittent theta-burst stimulation is attenuated in premanifest and early Huntington's disease. Sci. Rep. 12:1104. doi: 10.1038/s41598-021-04378-2, PMID: 35058470 PMC8776762

[ref2] Avan A. Digaleh H. Di Napoli M. Stranges S. Behrouz R. Shojaeianbabaei G.. (2019). Socioeconomic status and stroke incidence, prevalence, mortality, and worldwide burden: an ecological analysis from the global burden of disease study 2017. BMC Med. 17:191. doi: 10.1186/s12916-019-1397-3, PMID: 31647003 PMC6813111

[ref3] Badran B. W. Caulfield K. A. Cox C. Lopez J. W. Borckardt J. J. DeVries W. H.. (2020). Brain stimulation in zero gravity: transcranial magnetic stimulation (TMS) motor threshold decreases during zero gravity induced by parabolic flight. NPJ Microgravity 6:26. doi: 10.1038/s41526-020-00116-6, PMID: 33024819 PMC7505837

[ref4] Bejot Y. Bailly H. Durier J. Giroud M. (2016). Epidemiology of stroke in Europe and trends for the 21st century. Presse Med. 45, e391–e398. doi: 10.1016/j.lpm.2016.10.003, PMID: 27816343

[ref5] Bertani R. Melegari C. De Cola M. C. Bramanti A. Bramanti P. Calabro R. S. (2017). Effects of robot-assisted upper limb rehabilitation in stroke patients: a systematic review with meta-analysis. Neurol. Sci. 38, 1561–1569. doi: 10.1007/s10072-017-2995-5, PMID: 28540536

[ref6] Bertolucci F. Chisari C. Fregni F. (2018). The potential dual role of transcallosal inhibition in post-stroke motor recovery. Restor. Neurol. Neurosci. 36, 83–97. doi: 10.3233/RNN-170778 29439366

[ref7] Borrell J. A. Fraser K. Manattu A. K. Zuniga J. M. (2023). Laterality index calculations in a control study of functional near infrared spectroscopy. Brain Topogr. 36, 210–222. doi: 10.1007/s10548-023-00942-3, PMID: 36757503

[ref8] Bradnam L. V. Stinear C. M. Barber P. A. Byblow W. D. (2012). Contralesional hemisphere control of the proximal paretic upper limb following stroke. Cereb. Cortex 22, 2662–2671. doi: 10.1093/cercor/bhr344, PMID: 22139791 PMC4705341

[ref9] Buetefisch C. M. (2015). Role of the Contralesional hemisphere in post-stroke recovery of upper extremity motor function. Front. Neurol. 6:214. doi: 10.3389/fneur.2015.00214, PMID: 26528236 PMC4607877

[ref10] Chen N. Qiu X. Hua Y. Hu J. Bai Y. (2023). Effects of sequential inhibitory and facilitatory repetitive transcranial magnetic stimulation on neurological and functional recovery of a patient with chronic stroke: a case report and literature review. Front. Neurol. 14:1064718. doi: 10.3389/fneur.2023.1064718, PMID: 36779047 PMC9911674

[ref11] Chen C. Yuan K. Wang X. Khan A. Chu W. C. Tong R. K. (2021). Neural correlates of motor recovery after robot-assisted training in chronic stroke: a multimodal neuroimaging study. Neural Plast. 2021, 8866613–8866612. doi: 10.1155/2021/8866613, PMID: 34211549 PMC8208881

[ref12] Cunningham D. A. Machado A. Janini D. Varnerin N. Bonnett C. Yue G.. (2015). Assessment of inter-hemispheric imbalance using imaging and noninvasive brain stimulation in patients with chronic stroke. Arch. Phys. Med. Rehabil. 96, S94–S103. doi: 10.1016/j.apmr.2014.07.419, PMID: 25194451 PMC4348350

[ref13] de Freitas Z. A. Romeiro D. S. A. Baltar D. R. M. A. Shirahige G. D. N. L. Bezerra D. S. A. Piscitelli D.. (2022). Sensory and motor cortical excitability changes induced by rTMS and sensory stimulation in stroke: a randomized clinical trial. Front. Neurosci. 16:985754. doi: 10.3389/fnins.2022.985754, PMID: 36760794 PMC9907709

[ref14] Delorme M. Vergotte G. Perrey S. Froger J. Laffont I. (2019). Time course of sensorimotor cortex reorganization during upper extremity task accompanying motor recovery early after stroke: An fNIRS study. Restor. Neurol. Neurosci. 37, 207–218. doi: 10.3233/RNN-180877 31227675

[ref15] Di Lazzaro V. Pellegrino G. Di Pino G. Ranieri F. Lotti F. Florio L.. (2016). Human motor cortex functional changes in acute stroke: gender effects. Front. Neurosci. 10:10. doi: 10.3389/fnins.2016.00010, PMID: 26858590 PMC4731507

[ref16] Di Pino G. Pellegrino G. Assenza G. Capone F. Ferreri F. Formica D.. (2014). Modulation of brain plasticity in stroke: a novel model for neurorehabilitation. Nat. Rev. Neurol. 10, 597–608. doi: 10.1038/nrneurol.2014.162, PMID: 25201238

[ref17] Diao Q. Liu J. Wang C. Cao C. Guo J. Han T.. (2017). Gray matter volume changes in chronic subcortical stroke: a cross-sectional study. Neuroimage Clin. 14, 679–684. doi: 10.1016/j.nicl.2017.01.031, PMID: 28377881 PMC5369868

[ref18] Dionisio A. Duarte I. C. Patricio M. Castelo-Branco M. (2018). The use of repetitive transcranial magnetic stimulation for stroke rehabilitation: a systematic review. J. Stroke Cerebrovasc. Dis. 27, 1–31. doi: 10.1016/j.jstrokecerebrovasdis.2017.09.008 29111342

[ref19] Du J. Yang F. Hu J. Hu J. Xu Q. Cong N.. (2019). Effects of high- and low-frequency repetitive transcranial magnetic stimulation on motor recovery in early stroke patients: evidence from a randomized controlled trial with clinical, neurophysiological and functional imaging assessments. Neuroimage Clin. 21:101620. doi: 10.1016/j.nicl.2018.101620, PMID: 30527907 PMC6411653

[ref20] Du J. Yang F. Zhang Z. Hu J. Xu Q. Hu J.. (2018). Early functional MRI activation predicts motor outcome after ischemic stroke: a longitudinal, multimodal study. Brain Imaging Behav. 12, 1804–1813. doi: 10.1007/s11682-018-9851-y, PMID: 29766355

[ref21] Dum R. P. Strick P. L. (1991). The origin of corticospinal projections from the premotor areas in the frontal lobe. J. Neurosci. 11, 667–689. doi: 10.1523/JNEUROSCI.11-03-00667.1991, PMID: 1705965 PMC6575356

[ref22] Entakli J. Bonnard M. Chen S. Berton E. De Graaf J. B. (2014). TMS reveals a direct influence of spinal projections from human SMAp on precise force production. Eur. J. Neurosci. 39, 132–140. doi: 10.1111/ejn.12392, PMID: 24164635

[ref23] Gladstone D. J. Danells C. J. Black S. E. (2002). The Fugl-Meyer assessment of motor recovery after stroke: a critical review of its measurement properties. Neurorehabil. Neural Repair 16, 232–240. doi: 10.1177/154596802401105171, PMID: 12234086

[ref24] Groppa S. Oliviero A. Eisen A. Quartarone A. Cohen L. G. Mall V.. (2012). A practical guide to diagnostic transcranial magnetic stimulation: report of an IFCN committee. Clin. Neurophysiol. 123, 858–882. doi: 10.1016/j.clinph.2012.01.010, PMID: 22349304 PMC4890546

[ref25] Hallett M. Di Iorio R. Rossini P. M. Park J. E. Chen R. Celnik P.. (2017). Contribution of transcranial magnetic stimulation to assessment of brain connectivity and networks. Clin. Neurophysiol. 128, 2125–2139. doi: 10.1016/j.clinph.2017.08.007, PMID: 28938143 PMC5679437

[ref26] Huo C. Xu G. Li Z. Lv Z. Liu Q. Li W.. (2019). Limb linkage rehabilitation training-related changes in cortical activation and effective connectivity after stroke: a functional near-infrared spectroscopy study. Sci. Rep. 9:6226. doi: 10.1038/s41598-019-42674-0, PMID: 30996244 PMC6470232

[ref27] Kantak S. S. Stinear J. W. Buch E. R. Cohen L. G. (2012). Rewiring the brain: potential role of the premotor cortex in motor control, learning, and recovery of function following brain injury. Neurorehabil. Neural Repair 26, 282–292. doi: 10.1177/1545968311420845, PMID: 21926382 PMC4886541

[ref28] Kelley B. J. Harel N. Y. Kim C. Y. Papademetris X. Coman D. Wang X.. (2014). Diffusion tensor imaging as a predictor of locomotor function after experimental spinal cord injury and recovery. J. Neurotrauma 31, 1362–1373. doi: 10.1089/neu.2013.3238, PMID: 24779685 PMC4120934

[ref29] Kemlin C. Moulton E. Lamy J. C. Houot M. Valabregue R. Leder S.. (2019). Elucidating the structural and functional correlates of upper-limb Poststroke motor impairment. Stroke 50, 3647–3649. doi: 10.1161/STROKEAHA.119.027126, PMID: 31645211

[ref30] Kinoshita S. Tamashiro H. Okamoto T. Urushidani N. Abo M. (2019). Association between imbalance of cortical brain activity and successful motor recovery in sub-acute stroke patients with upper limb hemiparesis: a functional near-infrared spectroscopy study. Neuroreport 30, 822–827. doi: 10.1097/WNR.0000000000001283, PMID: 31283713

[ref31] Kumar P. Kathuria P. Nair P. Prasad K. (2016). Prediction of upper limb motor recovery after subacute ischemic stroke using diffusion tensor imaging: a systematic review and Meta-analysis. J. Stroke 18, 50–59. doi: 10.5853/jos.2015.01186, PMID: 26846758 PMC4747076

[ref32] Lam T. K. Binns M. A. Honjo K. Dawson D. R. Ross B. Stuss D. T.. (2018). Variability in stroke motor outcome is explained by structural and functional integrity of the motor system. Sci. Rep. 8:9480. doi: 10.1038/s41598-018-27541-8, PMID: 29930399 PMC6013462

[ref33] Lee S. H. Jin S. H. An J. (2019). The difference in cortical activation pattern for complex motor skills: a functional near- infrared spectroscopy study. Sci. Rep. 9:14066. doi: 10.1038/s41598-019-50644-9, PMID: 31575954 PMC6773684

[ref34] Lefaucheur J. P. Aleman A. Baeken C. Benninger D. H. Brunelin J. Di Lazzaro V.. (2020). Evidence-based guidelines on the therapeutic use of repetitive transcranial magnetic stimulation (rTMS): An update (2014-2018). Clin. Neurophysiol. 131, 474–528. doi: 10.1016/j.clinph.2019.11.002, PMID: 31901449

[ref35] Long H. Wang H. Zhao C. Duan Q. Feng F. Hui N.. (2018). Effects of combining high- and low-frequency repetitive transcranial magnetic stimulation on upper limb hemiparesis in the early phase of stroke. Restor. Neurol. Neurosci. 36, 21–30. doi: 10.3233/RNN-170733, PMID: 29439359

[ref36] Matsunaga K. Maruyama A. Fujiwara T. Nakanishi R. Tsuji S. Rothwell J. C. (2005). Increased corticospinal excitability after 5 Hz rTMS over the human supplementary motor area. J. Physiol. 562, 295–306. doi: 10.1113/jphysiol.2004.070755, PMID: 15513947 PMC1665472

[ref37] Najib U. Horvath J. C. (2014). “Transcranial Magnetic Stimulation (TMS) Safety Considerations and Recommendations,” in Transcranial Magnetic Stimulation, eds A. Rotenberg, J. Horvath, and A. Pascual-Leone (New York, NY: Humana Press), 89, 15–30. doi: 10.1007/978-1-4939-0879-0_2

[ref38] Ni J. Jiang W. Gong X. Fan Y. Qiu H. Dou J.. (2022). Effect of rTMS intervention on upper limb motor function after stroke: a study based on fNIRS. Front. Aging Neurosci. 14:1077218. doi: 10.3389/fnagi.2022.1077218, PMID: 36711205 PMC9880218

[ref39] Okabe N. Narita K. Miyamoto O. (2017). Axonal remodeling in the corticospinal tract after stroke: how does rehabilitative training modulate it? Neural Regen. Res. 12, 185–192. doi: 10.4103/1673-5374.200792, PMID: 28400791 PMC5361493

[ref40] Patel S. Ghimire P. Lavrador J. P. Jung J. Gullan R. Ashkan K.. (2020). Patient-reported experience measures in patients undergoing navigated transcranial magnetic stimulation (nTMS): the introduction of nTMS-PREMs. Acta Neurochir. 162, 1673–1681. doi: 10.1007/s00701-020-04268-y, PMID: 32100110 PMC7295840

[ref41] Plow E. B. Cunningham D. A. Varnerin N. Machado A. (2015). Rethinking stimulation of the brain in stroke rehabilitation: why higher motor areas might be better alternatives for patients with greater impairments. Neuroscientist 21, 225–240. doi: 10.1177/1073858414537381, PMID: 24951091 PMC4440790

[ref42] Prion S. Haerling K. A. (2014). Making sense of methods and measurement: Pearson product moment correlation coefficient. Clin. Simul. Nurs. 10, 587–588. doi: 10.1016/j.ecns.2014.07.005

[ref43] Shen Q. R. Hu M. T. Feng W. Li K. P. Wang W. (2022). Narrative review of noninvasive brain stimulation in stroke rehabilitation. Med. Sci. Monit. 28:e938298. doi: 10.12659/MSM.938298 36457205 PMC9724451

[ref44] Shirota Y. Hamada M. Terao Y. Ohminami S. Tsutsumi R. Ugawa Y.. (2012). Increased primary motor cortical excitability by a single-pulse transcranial magnetic stimulation over the supplementary motor area. Exp. Brain Res. 219, 339–349. doi: 10.1007/s00221-012-3095-7 22532164

[ref45] Stinear C. M. Petoe M. A. Byblow W. D. (2015). Primary motor cortex excitability during recovery after stroke: implications for neuromodulation. Brain Stimul. 8, 1183–1190. doi: 10.1016/j.brs.2015.06.015, PMID: 26195321

[ref46] Tamashiro H. Kinoshita S. Okamoto T. Urushidani N. Abo M. (2019). Effect of baseline brain activity on response to low-frequency rTMS/intensive occupational therapy in poststroke patients with upper limb hemiparesis: a near-infrared spectroscopy study. Int. J. Neurosci. 129, 337–343. doi: 10.1080/00207454.2018.1536053, PMID: 30311827

[ref47] Tang Q. Li G. Liu T. Wang A. Feng S. Liao X.. (2015). Modulation of interhemispheric activation balance in motor-related areas of stroke patients with motor recovery: systematic review and meta-analysis of fMRI studies. Neurosci. Biobehav. Rev. 57, 392–400. doi: 10.1016/j.neubiorev.2015.09.003, PMID: 26344667

[ref48] van Lieshout E. van der Worp H. B. Visser-Meily J. Dijkhuizen R. M. (2019). Timing of repetitive transcranial magnetic stimulation onset for upper limb function after stroke: a systematic review and Meta-analysis. Front. Neurol. 10:1269. doi: 10.3389/fneur.2019.01269, PMID: 31849827 PMC6901630

[ref49] Veldema J. Nowak D. A. Gharabaghi A. (2021). Resting motor threshold in the course of hand motor recovery after stroke: a systematic review. J. Neuroeng. Rehabil. 18:158. doi: 10.1186/s12984-021-00947-8, PMID: 34732203 PMC8564987

[ref50] Wang H. Arceo R. Chen S. Ding L. Jia J. Yao J. (2019). Effectiveness of interventions to improve hand motor function in individuals with moderate to severe stroke: a systematic review protocol. BMJ Open 9:e032413. doi: 10.1136/bmjopen-2019-032413, PMID: 31562163 PMC6773351

[ref51] Wang D. Wang J. Zhao H. Liang Y. Zhang W. Li M.. (2023). The relationship between the prefrontal cortex and limb motor function in stroke: a study based on resting-state functional near-infrared spectroscopy. Brain Res. 1805:148269. doi: 10.1016/j.brainres.2023.148269 36736871

[ref52] Xu Y. W. Lin P. Yao P. S. Zheng S. F. Kang D. Z. (2021). Structure and function of corticospinal projection originating from supplementary motor area. Neuroradiology 63, 1283–1292. doi: 10.1007/s00234-021-02669-z, PMID: 33611621

[ref53] Yang Y. Pan H. Pan W. Liu Y. Song X. Niu C. M.. (2021). Repetitive transcranial magnetic stimulation on the affected hemisphere enhances hand functional recovery in subacute adult stroke patients: a randomized trial. Front. Aging Neurosci. 13:636184. doi: 10.3389/fnagi.2021.636184, PMID: 34093164 PMC8171119

